# Maternal psychopathology and offspring clinical outcome: a four-year follow-up of boys with ADHD

**DOI:** 10.1007/s00787-016-0873-y

**Published:** 2016-07-04

**Authors:** Sharifah Shameem Agha, Stanley Zammit, Anita Thapar, Kate Langley

**Affiliations:** 1 Child and Adolescent Psychiatry Section, Division of Psychological Medicine and Clinical Neurosciences, School of Medicine, Cardiff University, Cardiff, Wales, UK; 2 Child and Adolescent Mental Health Services Network (CAMHS), Cwm Taf Health Board, Cardiff, Wales, UK; 3 MRC Centre for Neuropsychiatric Genetics and Genomics, School of Medicine, Cardiff University, Cardiff, Wales, UK; 4 School of Social and Community Medicine, University of Bristol, Bristol, UK; 5 School of Psychology, Cardiff University, Cardiff, Wales, UK

**Keywords:** ADHD, Conduct disorder symptoms, Mother self-reported ADHD, Mother self-reported depression

## Abstract

Previous cross-sectional research has shown that parents of children with attention deficit hyperactivity disorder (ADHD) have high rates of psychopathology, especially ADHD and depression. However, it is not clear whether different types of parent psychopathology contribute to the course and persistence of ADHD in the child over time. The aim of this two wave study was to investigate if mother self-reported ADHD and depression influence persistence of offspring ADHD and conduct disorder symptom severity in adolescents diagnosed with ADHD in childhood. A sample of 143 males with a confirmed diagnosis of ADHD participated in this study. ADHD and conduct disorder symptoms were assessed at baseline and reassessed 4 years later. The boys in this sample had a mean age of 10.7 years at Time 1 (SD 2.14, range 6–15 years) and 13.73 years at Time 2 (SD 1.74, range 10–17 years). Questionnaire measures were used to assess ADHD and depression symptoms in mothers at Time 1. Mother self-reported ADHD was not associated with a change in child ADHD or conduct symptom severity over time. Mother self-reported depression was found to predict an increase in child conduct disorder symptoms, but did not contribute to ADHD symptom levels. This study provides the first evidence that concurrent depression in mothers may be a predictor of worsening conduct disorder symptoms in adolescents with ADHD. It may, therefore, be important to screen for depression in mothers of children with ADHD in clinical practice to tailor interventions accordingly.

## Introduction

Attention deficit hyperactivity disorder (ADHD) is a disabling neurodevelopmental disorder that has major adverse consequences for individuals and their families [1]. Whilst previously considered as restricted to childhood, evidence indicates that ADHD persists into adulthood in 20–50 % of individuals [2, 3], and this persistence is associated with increased risk of additional problems including substance misuse, poor educational attainment, unemployment, friendship difficulties, and social problems [4, 5].

Despite its clinical importance, the causes and pathophysiology of ADHD are not well understood. It has been well established that ADHD is a highly familial and heritable disorder with mean heritability estimates of approximately 80 %, which also suggests that non-inherited factors also contribute [1]. The evidence to date indicates that ADHD is a complex multi-factorial disorder; that is a complex combination of many genes and non-inherited factors and their interplay all contribute to the aetiology of ADHD [6].

Parent psychopathology is known to be strongly associated with offspring disorder [7–10], although the genetic and environmental mechanisms that contribute to inter-generational links in psychopathology are complex [11]. Previous literature using case-control designs has shown evidence of higher rates of psychopathology, including ADHD and depression amongst parents (especially in mothers) of children with ADHD compared with parents of unaffected children [12–15]. Some studies have also shown the evidence of even higher rates of parent psychopathology in children with comorbid ADHD and oppositional defiant disorder or conduct disorder compared to children with ADHD alone [12, 16–19].

Within samples of children with ADHD, a number of cross-sectional studies, including our own [20], have demonstrated that parental ADHD is associated with a more severe clinical presentation of the disorder in offspring, including higher ADHD symptom severity [20–22], comorbid conduct disorder, and other psychopathology in general [20, 23]. There is also evidence that parental depression is associated with more severe clinical presentation and impairment in children [14, 23, 24] and that maternal psychopathology may be especially important [24]. Given these findings suggesting that parental psychopathology, and specifically ADHD and depression is associated with a more severe offspring ADHD clinical presentation, the next question is to what extent does parental psychopathology; specifically, maternal psychopathology longitudinally predicts (1) ADHD persistence and (2) long-term presence of conduct disorder symptoms in a clinical sample of children with ADHD?

There is some indication that parental psychopathology may be associated with persistence of ADHD longitudinally; one study found that family history of ADHD was associated with ADHD persistence [25]. Whether this was specific to mother’s or father’s ADHD was not reported, although the same group did find that maternal history of psychopathology (presence of at least two psychiatric disorders) predicts ADHD persistence [26]. Family history of mood disorders may also predict persistent ADHD [27], although an initial study within a community sample suggests that this may be due to paternal, rather than maternal mood [28]. These initial studies demonstrate that this is an interesting area of study, but that associations require further investigation to unpick the association of persistence of ADHD with parental ADHD and depression, especially looking at mothers and fathers separately. Given the previously discussed findings that parental psychopathology is associated with clinical presentation of ADHD in childhood, it is also important to investigate whether increased symptoms or persistence is a consequence of increased severity at diagnosis. In addition, recent treatment trials have shown emerging evidence that integrated intervention, including the treatment of mother depression and mother ADHD, is associated with improvements of mother symptoms, parenting and externalizing symptoms in samples of children with ADHD. Although still in its early stages, these findings indicate the importance of considering parent psychopathology in assessing treatment for children with ADHD [29, 30].

In the only study to date looking at the long-term outcomes for children with ADHD with depressed mothers, Chronis and colleagues found, as part of an eight-year longitudinal study of 108 families, that the children of mothers with depression have higher risk of developing conduct problems when controlling for baseline conduct severity [31]. This study did not look at the persistence of ADHD and mothers were asked about their lifetime history of depression. Therefore, it was not possible to tell if maternal depressive episodes had occurred during their child’s lifetime.

These initial studies indicate the potential importance of parental ADHD and depression as markers of ADHD prognosis in adolescence and potentially into adult life, although further work is needed to replicate these findings and investigate the area further. We aimed to address this by building upon previous work, including our own in a clinical sample of children with ADHD [20]. We used follow-up data to investigate whether mother self-reported ADHD and depression predicted persistence of ADHD and conduct disorder symptoms and diagnosis in adolescence, taking childhood ADHD severity into account. We hypothesized that mother self-reported ADHD and depression would be predictors of worse outcome, greater persistence, and less improvement in symptoms over time.

## Method

### Sample

The sample consisted of 143 males aged 10–17 years [mean age of 13.73 (SD 1.74)] diagnosed with ADHD in childhood, all of whom had participated in a larger genetics study approximately 4 years earlier (Time 1). Mean age at Time 1 was 10.71 years (SD 2.14) with an age range of 6–15 years. Participants were initially recruited from child and adolescent psychiatry and pediatric services in the UK. Each child met diagnostic criteria for ADHD confirmed by research diagnostic interviews at Time 1 (see [32] for further details). Children with any known diagnosis of schizophrenia, autism spectrum disorder (ASD), bipolar disorder, Tourette’s syndrome, epilepsy, brain damage or any other neurological or genetic disorder were excluded from the study. Male participants aged between 10 and 17 years with an IQ > 70 were invited to take part at Time 2 (IQ < 70 was an exclusion criteria only for the follow up part of this study). Amongst those invited to take part at Time 2, 72 % agreed to participate. The mean time between the two assessments was 2.59 years (SD 0.91), range of 1–5 years in between Time 1 and Time 2.

Informed written consent and assent were obtained from parents and young people at both time points. Ethical approval for the study was obtained from the Wales Multicentre Research Ethics Committee.

### Measures

#### Child psychopathology

Child psychopathology at Time 1 was assessed using the Child and Adolescent Psychiatric Assessment (CAPA) [33], a semi-structured research diagnostic interview (see [34] for further details). Child psychopathology at Time 2 was re-assessed using the Development and Well-being Assessment (DAWBA) structured interview [35]. The DAWBA was used to reduce the assessment time burden on families. At both time points, parents completed the ADHD and Conduct Disorder (CD) sections and young people the CD section. Conduct disorder symptoms were rated as present when endorsed by either the parent or young person. All interviews were administered by trained psychologists supervised weekly by a child psychiatrist (AT) and a psychologist (KL). Symptom scores and diagnoses were generated from the CAPA and DAWBA. The original study used DSM-IV criteria. For the purpose of this longitudinal analysis, DSM-5 criteria were used to define Time 1 and Time 2 ADHD and CD symptom scores and diagnosis. Young people were defined as having persistent ADHD if they met DSM-5 diagnostic criteria for ADHD at both Time 1 and Time 2, and remitted ADHD if they did not meet diagnostic criteria for ADHD at Time 2. As different assessment tools were used at Time 1 (CAPA) and Time 2 (DAWBA), the symptom scores for ADHD and conduct disorder were standardized and used in all the analyses.

Information on whether young people had a current prescription for ADHD medication was also obtained. At Time 1, 83 % of the children were treated on ADHD medication and 80 % at Time 2. There was no information collected on any non-pharmacological treatments.

#### Maternal psychopathology

Maternal psychopathology was assessed at Time 1 using questionnaire measures. Mothers completed a questionnaire regarding ADHD symptoms in themselves at age 7–11 years (childhood) and in the last 6 months (current), using an 18 item checklist of DSM-5 ADHD symptoms (see [20] for further details). Total scores were generated separately for childhood and current symptoms. Positive ADHD status was assigned if symptom criteria were met for a DSM-5 ADHD diagnosis (a minimum of six inattentive or hyperactive/impulsive symptoms in childhood and at least five inattentive or hyperactive/impulsive symptoms at present).

To measure parental depression, mothers completed the Hospital Anxiety and Depression Scale (HADS) at Time 1 [36]. As in previous validation studies, a cut-off score of 11 or higher was used to indicate the presence of a mood disorder based on 7 depression items from the HADS [37, 38].

Mothers also completed a DSM-5 conduct symptom checklist on the presence of conduct disorder symptoms in themselves at age 7–11 years. Symptom presence was rated on a Likert scale from 0 to 3 (‘never’, ‘rarely’, ‘sometimes,’ and ‘often’). Ratings of ‘sometimes’ or ‘often’ were taken to indicate the presence of a symptom which were then summed to calculate a total symptom score of mother self-reported conduct symptoms in childhood.

#### ADHD and CD symptom change

ADHD symptom and conduct symptom change scores were calculated to observe the changes in these symptoms over time. Total symptoms at Time 2 were subtracted from total symptoms at Time 1. Negative scores indicate symptom reduction over time, and positive scores indicate increase over time.

### Statistical analysis

Mother self-reported ADHD and depression were considered as predictors using both binary [presence of ADHD diagnosis status (meeting DSM-5 criteria during childhood and current ADHD) and presence of mother depression (using HADS cut-point) and dimensional scores (mother self-reported current ADHD symptoms and depression symptom scores]. Offspring ADHD outcome measures were (1) ADHD diagnosis at Time 2—persistent and remitted ADHD, (2) ADHD change score, and (3) ADHD symptom severity at Time 2. Offspring CD outcome measures were (1) CD change score and (2) CD symptom severity at Time 2.

Logistic regression was used to estimate odds ratios and 95 % confidence intervals to predict ADHD persistence in the child at Time 2 in relation to maternal psychopathology. Linear regression was used to estimate differences (and 95 % confidence intervals) in child ADHD and conduct symptom severity scores at Time 2 and change in symptom scores from Time 1 to Time 2 in relation to maternal psychopathology at Time 1. We adjusted for child age, child ADHD and conduct disorder symptoms at Time 1, mother self-reported childhood conduct symptoms, period between Time 1 and Time 2, and ADHD medication status at Time 2 as potential confounders in the final model. All analyses were performed using STATA (version13).

## Results

Clinical and demographic data were compared between families who took part at both time points and those participants recruited at Time 1 only. There were no differences between these groups in ADHD and conduct symptom severity or prevalence of mother self-reported depression and ADHD at Time 1. In this sample, we found that 19 % (95 % CI 0.13–0.27) of mothers met the study criteria for ADHD and 21 % (95 % CI 0.14–0.28) of mothers met the study criteria for depression. Only 6.6 % of mothers met study criteria for both ADHD and depression. Sociodemographic characteristics of the sample are shown in Table 1.

**Table 1:** Sociodemographic characteristics of the sample

	*n*	Mean (SD)/%
Child age Time 1 (years)	143	10.71 (2.14)
Child age Time 2 (years)	143	13.73 (1.74)
Maternal age at Time 1	140	36.72 (5.93)
Single parent household	81	57 %
Low social class^a^	52	41 %
Low parent education^b^	26	20 %
Low income^c^	76	59 %

^a^Low social class: occupation of main family wage earner as unskilled worker/unemployed, classified using the UK Standard Occupation Classification 2000. ^b^ Low parent education: left school without qualifications (GCSE or equivalent). ^c^ Low income: annual family income <£20,000 (equivalent ~$32,000)

At Time 2, 82 % (*n* = 112) of the young people continued to meet full criteria for DSM-5 ADHD diagnosis—persistent ADHD (mean symptoms 14.29, SD 2.87). The remaining 18 % (*n* = 25) of young people did not meet full diagnostic ADHD criteria at Time 2—remitted ADHD. Although they no longer met criteria for ADHD, these young people still had ADHD symptoms (mean 5.76, SD 2.77) and 19 young people were still being treated with ADHD medication.

At Time 1, 20 % of young people had a DSM-5 diagnosis of conduct disorder [mean symptom score 1.30 (SD 1.65)]. The prevalence of conduct disorder at Time 2 was 53 % (mean symptoms 3.48, SD 3.07). A total of 36 % of young people had developed new onset conduct disorder at Time 2 and 17 % had conduct disorder persisting between both time points. Only 3 % of those with conduct disorder at Time 1 no longer fulfilled symptom criteria at Time 2 (mean 1.17, SD 0.41).

### Mother self-reported ADHD

Mother self-reported ADHD status did not predict ADHD persistence in adolescents [OR 1.16 (0.36, 3.78) *p* = 0.80]. There was also no evidence of association between mother self-reported ADHD status and child ADHD symptom severity at Time 2 (*β* = 0.22, 95 % CI −0.16, 0.59, *p* = 0.25) (Table 2). In relation to symptom change, although mean ADHD symptom score change was lower over time amongst young people who had a mother with ADHD compared to those without (−1.88 vs −2.66, respectively) this difference was not statistically significant (*p* = 0.35) (Table 3).

**Table 2:** Associations between maternal psychopathology and (a) child ADHD symptoms at Time 2 and (b) child conduct symptoms at Time 2

	Model 0			Model 1			Model 2		
	β	(95 % CI)	p	β	(95 % CI)	p	β	(95 % CI)	p
(a) Child ADHD symptoms at Time 2^a^									
Mother self-reported ADHD	0.23	(−0.19, 0.66)	0.278	0.16	(−0.20, 0.52)	0.383	0.22	(−0.16, 0.59)	0.249
Mother ADHD current symptoms^+^	0.10	(−0.07, 0.27)	0.237	0.07	(−0.07, 0.22)	0.316	0.09	(−0.06, 0.23)	0.252
Mother self-reported depression	0.18	(−0.23, 0.59)	0.381	0.003	(−0.35, 0.36)	0.984	−0.02	(−0.39, 0.35)	0.912
Mother depression symptoms^+^	0.07	(−0.09 0.24)	0.384	−0.04	(−0.18, 0.11)	0.601	−0.03	(−0.18, 0.12)	0.682
(b) Child conduct symptoms at Time 2^b^									
Mother self-reported ADHD	0.23	(−0.22, 0.68)	0.319	0.24	(−0.16, 0.65)	0.236	0.11	(−0.34, 0.55)	0.635
Mother ADHD current symptoms^+^	0.14	(−0.03, 0.32)	0.110	0.17	(0.01, 0.33)	0.036*	0.15	(−0.02, 0.33)	0.088
Mother self-reported depression	0.78	(0.37, 1.20)	<0.001*	0.54	(0.14, 0.93)	0.008*	0.47	(0.05, 0.88)	0.027*
Mother depression symptoms^+^	0.34	(0.17, 0.51)	<0.001*	0.26	(0.10, 0.42)	0.001*	0.24	(0.07, 0.41)	0.005*

^a^ Model 0: Unadjusted. Model 1: Adjusted for ADHD severity Time 1 (standardised score). Model 2: Adjusted for ADHD severity Time 1 (standardised score) period between Time 1 & 2, ADHD medication Time 2, child age

^b^ Model 0: Unadjusted. Model 1: Adjusted for CD severity Time 1 (standardised score). Model 2: Adjusted for CD severity Time 1 (standardised score), maternal childhood CD symptoms, period between Time 1 & 2, ADHD medication Time 2, child age

* *p* < 0.05

^+^ Standardised score

**Table 3:** Mean scores (a) child ADHD symptom change scores and (b) child conduct symptom change

	(a) Child ADHD symptom change Mean (sd)	(b) Child conduct symptom change Mean (sd)
Mother ADHD		
None	−2.66 (3.80)	2.13 (2.80)
Present	−1.88 (3.63)	2.84 (3.10)
Mother depression		
None	−2.52 (3.87)	1.97 (2.69)
Present	−2.54 (3.37)	3.36 (3.25)*

* *p* < 0.05

With regard to conduct disorder symptoms, there was no evidence of an association between mother self-reported ADHD status and conduct symptom severity at Time 2 (*β* = 0.11, 95 % CI −0.34, 0.55, *p* = 0.64) (Table 2) and with conduct change score (*β* = 0.71, 95 % CI −0.54, 1.96, *p* = 0.26). However, there was a trend towards an association between mother current ADHD symptoms and child conduct symptom severity (*β* = 0.15, 95 % CI −0.02, 0.33, *p* = 0.09).

In this sample, we did not find associations between mother self-reported ADHD status and child ADHD and conduct symptoms at Time 1, even though we had found these associations previously [20]. This could be due to smaller sample size compared to the study at Time 1.

### Mother self-reported depression

Mother self-reported depression status did not predict ADHD diagnostic persistence in adolescents [OR 1.94 (0.53, 7.08), *p* = 0.32)]. It was also found that depression status in mothers did not predict child ADHD severity at Time 2 or ADHD symptom change score (Tables 2, 3). At Time 1, mother self-reported depression status was associated with conduct disorder symptoms and there was weak evidence of an association with child ADHD symptoms.

Mother self-reported depression status was associated with conduct symptom severity at Time 2, and this persisted after controlling for severity of child conduct disorder symptoms at Time 1. (*β* = 0.54, 95 % CI 0.14, 0.93, *p* = 0.008) (Table 2). We also found that mean conduct disorder change score was significantly greater in offspring of mothers with depression compared to those whose mothers did not have depression (3.36 vs 1.97, *p* = 0.02) (see Table 3).

Several probable confounders were identified which could potentially influence the association between mother self-reported depression and child conduct outcome at Time 2. The period between Time 1 and Time 2, mother childhood conduct disorder symptoms, and ADHD medication status at Time 2 were found to be correlated with mother depression status and child CD outcome at Time 2. After adjusting for child age and the confounders mentioned above, the effect of mother self-reported depression status on child conduct outcome at Time 2 was slightly attenuated, but the association still remained (*β* = 0.47, 95 % CI 0.05, 0.88, *p* = 0.03) (Table 3). Similar results were obtained for associations with mother depression symptoms (*β* = 0.24, 95 % CI 0.07, 0.41, *p* = 0.005). Adjusting for baseline medication and oppositional defiant disorder symptoms at Time 1 did not alter the association between mother depression status and child conduct disorder.

### Further analysis with social class as covariate

As a separate analysis, we examined to what extent all observed associations changed after adjustment for low social class. Adjusting for low social class had slightly attenuated associations between mother self-reported depression and child conduct symptoms at Time 2 by approximately 6–12 % [mother self-reported depression status (*β* = 0.44, 95 % CI −0.02, 0.90, *p* = 0.06) and mother depression symptoms (*β* = 0.21, 95 % CI 0.02, 0.39, *p* = 0.027)].

## Discussion

In the present study, we investigated whether mother self-reported ADHD and depression predicted clinical outcome of adolescent boys with ADHD across 4 years. High prevalence of maternal psychopathology was observed at Time 1, where 19 % (95 % CI 0.13–0.27) of mothers met study criteria for ADHD (DSM-5) and 21 % (95 % CI 0.15–0.28) of mothers met the cut-point for depression. These rates are much higher compared to results from a national comorbidity survey of adults in the general population which reported that 3–5 % of mothers had ADHD and 12.9 % had depression in the past year, although our measures are questionnaire-based [39]. The prevalence of ADHD and depression in mothers in this study is similar and slightly higher to other studies with a sample of children with ADHD or other psychiatric disorders [14, 41, 41].

Looking at clinical outcome at Time 2, there was a high prevalence of young people who still met full DSM-5 diagnostic criteria of ADHD. The pattern of symptom change over time was as expected; ADHD symptoms reduced with age and CD symptoms increased in adolescence. However, the prevalence of ADHD and conduct disorder highlights the fact that these young people are still very much symptomatic and impaired.

Contrary to our hypothesis, we did not find an association between mother self-reported ADHD and the course or persistence of clinical symptoms of ADHD or conduct disorder across adolescence, even though we had previously found associations between mother self-reported ADHD and these clinical measures cross sectionally at Time 1 [20]. However, there was a trend for an association between mother current ADHD symptoms and child conduct disorder symptoms. In addition, mean ADHD symptom change was lower in those with a mother with ADHD, which implies that this group showed less improvement of ADHD symptoms over time, but we may have been underpowered to detect any effects of this size.

We did, however, find that mother self-reported depression influences the later development of conduct disorder symptoms in adolescent boys with ADHD, which is consistent with our hypothesis. The results of the present study are in keeping with the previous findings from Chronis et al., where it was found that history of maternal depression predicted later development of conduct problems [31]. Our study extends these findings by investigating the influences of mother self-reported ADHD and concurrent mother self-reported depression.

Unlike the findings from Biederman [25–27] on ADHD persistence, maternal psychopathology in this study was not found to predict ADHD persistence. These differences could be due to the inclusion of a broad range of psychopathology on any first degree relative in the studies by this group, including fathers and siblings [25, 27] or the lack of specificity regarding maternal diagnoses [26]. Therefore, the previous findings are perhaps not specific to mother self-reported depression or ADHD. In this regard, our findings do concur with those of Lara and colleagues who did not find an association between maternal mood and anxiety and ADHD persistence [28]. In addition, differences in defining ADHD using DSM-5 may have contributed as well, although similar results were found within this sample when defining ADHD using DSM IV criteria. Our rates of ADHD persistence were also high, possibly because the follow-up period was only after 4 years. This needs further investigation in a larger sample of young people over a longer period of time.

Parents play a significant role in providing the caregiving environment and having the earliest influences on a child’s development. The association between depression in parents and child ADHD is likely to have come about for a variety of reasons. Parents of children with ADHD are at heightened genetic risk of depression and experience chronic stress from their children’s symptoms and from economic strain which are potent risk factors for depression [42, 43]. Parenting difficulties and the quality of parent–child relationship could also be another possible mechanism which might explain the link between depression in mothers and conduct disorder [44]. One study suggests that responsiveness in parenting acts as a mediating mechanism in the relationship between parent depressive symptoms and conduct problems in children with ADHD [45]. Several studies in families of children with ADHD have found that currently depressed mothers face more parenting challenges relative to non-depressed mothers and that they are more susceptible to child characteristics which can affect the quality of parent–child relationships [46–48].

Another explanation is direct child effects on parent. A recent adoption design suggests the importance of child ADHD on mother–child relationship, where genetically influenced child ADHD characteristics elicit hostility in parenting [49]. Treatment studies have shown that mother–child relationships improve the following treatment of child ADHD symptoms [50].

Paternal psychopathology is another important consideration. ADHD is more common in males and we cannot rule out the possibility that the association between mother self-reported depression and child conduct problems is explained by paternal mental health as well as other unmeasured confounders. Like most observational studies, genetic factors may also contribute to residual confounding.

As there were high rates of families in this sample with low social class (41 %), we investigated if any observed associations were confounded by having a lower socioeconomic status. Comparison of estimates showed that the associations were only slightly attenuated by about 6–12 %, and associations between mother depression symptoms and child conduct severity at Time 2 remained significant. This indicates that even if low social class status may be a confounder, the associations were not completely attenuated by adjustment for this. Furthermore, it is not possible to distinguish whether social class is a confounder or acts as a mediator of the relationship between parent psychopathology and child outcome. For example, it is feasible that parents can end up in a lower social class as a result of having functional impairments related to ADHD or depression which lowers their ability to achieve both educationally and occupationally. Consequently, growing up in this environment could increase the severity of ADHD in offspring (e.g., insufficient resources or support). However, as for any observational study, we are unable to exclude the possibility of residual confounding, for example, by other characteristics associated with parent ADHD or depression.

To our knowledge, this is the first study investigating the different influences of mother self-reported ADHD and concurrent depression in mothers on future outcomes in a clinical sample of boys with ADHD taking baseline symptoms into account. The study looks at symptom change over time and includes measures of child and mother psychopathology using DSM-5 criteria. It also takes into account both mother and child reports of child conduct disorder symptoms.

This study, however, should be considered in view of certain limitations. First, we could not look at the effects of paternal psychopathology, as there was insufficient data available from fathers. Many families ascertained in this sample were single parent families (mostly mothers), and there is evidence to suggest that the inclusion of only intact families may result in a sample with a less severe clinical presentation of ADHD [51]. In the analyses, whilst we controlled for current medication use of the children, we did not have information on any psychological or non-pharmalogical treatments.

Unfortunately, there was no measure of child mood or anxiety problems at follow-up, and so, we were unable to investigate the outcome of these disorders in this sample. There was also no current measure of maternal psychopathology at Time 2; therefore we were unable to test specific timing effects of depression in mothers in relation to child disorder. In addition, it is not possible to rule out measurement error effects that might have biased any findings in relation to mother self-reported ADHD or depression and change in child outcome over time.

Depression status for mothers in this study was obtained from the HADS which was initially developed for screening purposes and, therefore, does not represent definitive diagnosis of depression. However, the HADS has been widely used, reported to have good validity and performs well in predicting caseness of anxiety disorder and depression in both psychiatric and primary care patients as well as the general population [38]. Questionnaire measures of parent mental health are also likely to be more practical in settings that focus primarily on child mental health. There are concerns that parental depression can bias the reporting of child behavior. However, the measure of conduct disorder symptoms here includes child self-reports, and there has also been evidence to suggest that parents with depression can reliably report on child psychopathology and behavior [52–54].

Another limitation is that child psychopathology at Time 2 was assessed using the DAWBA, a structured interview, which was different to assessment at Time 1 where the CAPA, a semi structured interview, was used. However, a study comparing three different psychiatric interviews, including the DAWBA and CAPA, found that the DAWBA reported similar rates of ADHD and CD as the CAPA [55]. To account for this, standardized scores were used in the analyses.

Adults with ADHD are reported to have high rates of comorbid anxiety and depression [56]. It would have been interesting to investigate the influence of comorbid parent psychopathology. However, there was little overlap in this sample of mothers who had both ADHD and depression [6.6 % (*n* = 9)], and therefore, there was insufficient power to further investigate this. Mothers of children with ADHD are also reported to have higher anxiety symptoms compared to controls [22]. In this sample, there was considerable overlap observed between some of the anxiety items of the HADS questionnaire and ADHD symptoms, such as restlessness and ‘being on the move’. Therefore, it was decided that this questionnaire measure of anxiety might not be valid in parents of children with ADHD. This would, however, be interesting to study in the future.

This analysis was conducted on a sample of boys, and therefore, we could not investigate if the effect of parental psychopathology on child outcomes would be the same in sample of girls with ADHD. The previous studies have found that girls are more sensitive or vulnerable to effects of maternal depression as they enter adolescence compared to boys [57–59]. The findings and conclusions from this study are specific to young adolescent boys with ADHD. Future research should consider investigating the differences of effect on boys and girls.

Findings from the present study have important clinical implications. When assessing children with ADHD in clinic, it is important for clinicians to be aware of the high prevalence of parent mental health problems. It may be especially important to screen for depression in mothers and tailor treatment and intervention accordingly. It is also important to consider the multiple impairments or difficulties faced by families, especially if the parent has mental health difficulties [60]. Preliminary evidence in a recent trial, revealed that an integrated intervention-based treatment incorporating parenting training and cognitive behavioral depression treatment had slightly better beneficial effects compared to parenting training alone in a sample of children with ADHD [29]. Treatment of parent depression in randomized controlled trials have also been found to result in improvements in child mental health, especially conduct problems [61].

Overall, our results suggest that concurrent depression in mothers predicts adverse clinical outcome in terms of conduct disorder symptoms in a sample of boys with ADHD. Further work is needed to understand the processes that contribute to this link, given the global impairment in functioning associated with conduct disorder in ADHD. The study also suggests that the influence of parent psychopathology on longer term outcomes in boys with ADHD may differ by specific parental psychopathology. However, we are not able to test this directly due to our limited sample size, and this needs to be investigated further.
